# Supplementary material for: Multiplexed detection of RNA using MERFISH and branched DNA amplification
Source: Sci Rep. 2019 May 22;9:7721. doi: 10.1038/s41598-019-43943-8 (PMC6531529; doi:10.1038/s41598-019-43943-8)
Supplement: Supplementary file 1 — Supplementary Info [file 41598_2019_43943_MOESM1_ESM.docx]

Supplementary Information for

**Multiplexed detection of RNA using MERFISH and branched DNA amplification**

Chenglong Xia^1^, Hazen P. Babcock^2^, Jeffrey R. Moffitt^3^, Xiaowei Zhuang^1^

^1^Howard Hughes Medical Institute, Department of Chemistry and Chemical Biology, and Department of Physics, Harvard University, Cambridge, MA 02138**,** USA

^2^Center for Advanced Imaging, Harvard University, Cambridge, MA 02138**,** USA

^3^Program in Cellular and Molecular Medicine, Boston Children’s Hospital; Department of Microbiology, Harvard Medical School, Boston, MA 02115, USA

Supplementary Information includes:

Caption for Supplementary Table 1.

**Table S1**: Sequences of the amplifier oligonucleotides and readout sequences used for MERFISH measurements (provided as a separate xls file). “Bit” is the number of the bit in the barcode. “Original readout sequences” are the sequences of the original readout sequence associated with each bit prior to amplification. “Primary amplifier sequences” are the sequences of the primary amplifier molecules corresponding to each bit. “Secondary amplifier sequences” are the sequences of the secondary amplifier molecules corresponding to each bit. “Final readout sequences” are the sequences of the fluorescently labeled readout probes used to measure the corresponding bit after amplification.
